# Low-Resolution Place and Response Learning Capacities in Down Syndrome

**DOI:** 10.3389/fpsyg.2018.02049

**Published:** 2018-10-26

**Authors:** Mathilde Bostelmann, Floriana Costanzo, Lorelay Martorana, Deny Menghini, Stefano Vicari, Pamela Banta Lavenex, Pierre Lavenex

**Affiliations:** ^1^Laboratory of Brain and Cognitive Development, Institute of Psychology, University of Lausanne, Lausanne, Switzerland; ^2^Department of Neuroscience, Bambino Gesù Children’s Hospital, Rome, Italy

**Keywords:** allocentric, egocentric, spatial memory, multiple memory systems, Down syndrome, dissociation

## Abstract

Down syndrome (DS), the most common genetic cause of intellectual disability, results from the partial or complete triplication of chromosome 21. Individuals with DS are impaired at using a high-resolution, allocentric spatial representation to learn and remember discrete locations in a controlled environment. Here, we assessed the capacity of individuals with DS to perform low-resolution spatial learning, depending on two competing memory systems: (1) the place learning system, which depends on the hippocampus and creates flexible relational representations of the environment; and (2) the response learning system, which depends on the striatum and creates fixed stimulus–response representations of behavioral actions. Individuals with DS exhibited a preservation of the low-resolution spatial learning capacities subserved by these two systems. In place learning, although the average performance of individuals with DS was lower than that of typically developing (TD) mental age (MA)-matched children and TD young adults, the number of individuals with DS performing above chance level did not differ from TD children. In response learning, the average performance of individuals with DS was lower than that of TD adults, but it did not differ from that of TD children. Moreover, the number of individuals with DS performing above chance level did not differ from TD adults, and was higher than that of TD children. In sum, whereas low-resolution place learning appears relatively preserved in individuals with DS, response learning appears facilitated. Our findings are consistent with the hypothesis that the neural pathways supporting low-resolution place learning and response learning are relatively preserved in DS.

## Introduction

Down syndrome (DS), resulting from a partial or complete triplication (trisomy) of chromosome 21, is the most common genetic cause of intellectual disability, with an incidence of 1 in 625–1,000 live births (Bittles et al., 2006; de Graaf et al., 2017). Adults with DS have IQs ranging from 30 to 70 and a typical mental age (MA) ranging from 5 to 9 years of age (Vicari et al., 2005, 2006). Despite a global mental retardation, individuals with DS show a unique cognitive profile compared to other genetic disorders. Individuals with DS exhibit specific difficulties in the verbal domain, including poor language abilities (Chapman et al., 1991; Chapman, 1997; Abbeduto et al., 2003) and impairments in verbal short-term memory (Jarrold and Baddeley, 1997), especially in maintaining phonological information over a short delay (Raitano Lee et al., 2010). By contrast, their visuo-spatial memory (“*where*” *memory*) is reported as relatively preserved. For example, individuals with DS exhibit a performance similar to that of MA-matched typically developing (TD) children in learning where pictures are presented on a piece of paper (Vicari et al., 2005) and on the Corsi block-tapping task (Wang and Bellugi, 1994; Jarrold and Baddeley, 1997; Numminen et al., 2001; Laws, 2002). However, not all non-verbal capacities are spared. Individuals with DS tend to neglect the internal details of stimuli, such as in the Delis hierarchical processing task, and instead exhibit a bias toward the global features of those stimuli (Bellugi et al., 1999), but see D’Souza et al., 2016. Difficulties in memorizing and recognizing pictures of objects (“*what*” *memory)* have also been reported in DS (Vicari, 2001; Vicari et al., 2005).

Germane to the current study, previous investigations of spatial memory in DS mainly assessed small-scale visuo-spatial capacities. Research has shown, however, that performance on small-scale spatial tasks do not correlate with performance on large-scale spatial tasks, particularly those in which participants must move around (Quaiser-Pohl et al., 2004; Hegarty et al., 2006; Farran et al., 2010). Evaluating the large-scale spatial capacities of individuals with DS is important because spatial deficits may limit their everyday functioning and autonomy. Nevertheless, when evaluating spatial learning and memory capacities, it is important to consider the influence that other cognitive processes, including but not limited to, verbal comprehension (of task rules, etc.), working memory, executive functions, and visual imagery, may have on performance. Moreover, it is fundamental to recognize that there are different types of dissociable spatial memory systems that may interact and contribute to guiding behavior, and thus impact overall task performance (Banta Lavenex and Lavenex, 2009; Banta Lavenex et al., 2015; Bostelmann et al., 2017).

In the current study, we investigated the ability of individuals with DS to rely on two different spatial memory systems in order to identify one discrete location among four possible locations in a controlled environment: (1) the place learning system, responsible for creating allocentric spatial representations or cognitive maps, and which has been shown to depend on the integrity of the hippocampus in rats, monkeys, and humans (O’Keefe and Dostrovsky, 1971; O’Keefe and Nadel, 1978; Morris et al., 1982; Banta Lavenex et al., 2006; Banta Lavenex et al., 2014); and (2) the response learning system, responsible for creating fixed stimulus–response representations of behavioral actions also known as habits, and which has been shown to depend on the dorsal striatum in rats and humans (Packard et al., 1989; Packard and McGaugh, 1996; Yin and Knowlton, 2006).

### Place Learning

Place learning refers to an individual’s ability to learn and remember locations in an allocentric spatial frame of reference. In this representation, locations are encoded in relation to other objects or locations in the environment (i.e., in a viewpoint-independent manner), thus enabling the creation of a cognitive map of the environment (Tolman, 1948; O’Keefe and Nadel, 1978; Banta Lavenex et al., 2014). For example, imagine a teacher’s desk that can be described in allocentric terms as both in front of the class and in the center of the room; importantly, the desk maintains its relations to other fixed objects irrespective of where a student is sitting in the classroom. The ability of individuals with DS to use place learning has been previously assessed in virtual environments as a specific test for “hippocampus-dependent” memory. For example, Pennington et al. (2003) employed a virtual Morris water maze task, a classic task for investigating place learning in rodents (Morris et al., 1982), in which individuals had to learn the position of a target within a virtual room containing distal visual cues. During a probe trial without the actual target, individuals with DS spent less time searching in the vicinity of the target location, as compared to TD children, thus suggesting an impairment in hippocampus-dependent spatial learning in DS. However, in a subsequent study including a larger group of participants, Edgin et al. (2010) failed to find a difference between individuals with DS- and MA-matched TD children, raising questions about the usefulness or reliability of this task to characterize the spatial cognitive profile of individuals with DS.

In another virtual reality study, Courbois et al. (2013) tested the ability of 10 individuals with DS to take a novel route (i.e., shortcut between two previously traveled routes), as a means of assessing their ability to build a cognitive map. Seven of the individuals with DS were able to learn two initial routes (i.e., three did not), by either memorizing ordered sequences of landmarks and actions to be taken (e.g., go to the bridge and turn right, then go to the tower and turn left, etc.) or by using a beacon-following strategy moving toward a series of landmarks (e.g., go toward the bridge, look around and find the tower, then go toward the tower, etc.). A third possible strategy not considered by the authors was for participants to learn a sequence of turns (see response learning, below). Eventually, only two of the seven individuals with DS who had learned the routes were able to take a shortcut between the start and end locations. Overall, these results suggest that individuals with DS may have relatively preserved route learning abilities, but greater difficulty in representing the configural or spatial relationships between landmarks constituting the environment, i.e., building an allocentric spatial representation of the environment that subserves successful place learning.

Although spatial tasks conducted in virtual reality tend to approximate real-world tasks more closely than standard tabletop neuropsychological tests, their ethological validity has been questioned (Banta Lavenex and Lavenex, 2009; Taube et al., 2013). In the real world, different sensory modalities (including visual, vestibular, and proprioceptive information) provide information that is coherent. This information is integrated by the brain, including the hippocampus, to construct reliable representations of an individual’s experiences. By contrast, in virtual reality studies, different inputs derived from different sensory modalities are often incoherent. In that context, Banta Lavenex et al. (2015) investigated the place learning capacities of adult individuals with DS- and MA-matched TD children in a spatial learning task carried out in a real-world, controlled environment. Participants had to locate three rewards among 12 potentially rewarded locations arranged on three nested square arrays within a 4 m × 4 m testing arena. Individuals with DS made fewer correct choices before erring, visited more locations to find the three rewards, and performed fewer errorless trials than MA-matched TD children. However, task performance varied among individuals with DS, and 50% of the individuals with DS performed above chance level. Interestingly, these individuals were able to choose preferentially the rewarded location located on the outer array, which could be identified using a low-resolution topological representation of the environment (Poucet and Benhamou, 1997). Only two individuals with DS (out of 20) were able to reliably identify the other two rewarded locations located on the middle and inner arrays, which required the ability to build a high-resolution spatial representation of the environment. These previous results thus suggest that low-resolution place learning may be relatively preserved in individuals with DS (e.g., as compared to TD children), whereas high-resolution place learning may be more impacted (e.g., as compared to TD children). Data in rodents and humans suggest that low-resolution topological coding and high-resolution metric coding implicate different hippocampal circuits, respectively, the CA1 field of the hippocampus and the dentate gyrus-CA3 field (Gilbert et al., 2001; Gilbert and Kesner, 2006; Lavenex and Banta Lavenex, 2013). Our behavioral data in individuals with DS therefore suggest that the dentate gyrus-CA3 functional circuit may be more systematically impaired, whereas CA1 function may be significantly impaired in only about half of individuals with DS. However, because participants had to remember three locations among 12 possible locations, it is not clear whether task performance was also impacted by the number of locations to be remembered (i.e., memory load). Consequently, it remains to be determined whether, as a group, individuals with DS are able to perform place learning for a single location, using a low-resolution topological representation of the environment.

### Response Learning

The response learning system creates fixed stimulus–response representations of behavioral actions, also known as habits (Packard and McGaugh, 1996). This system has been shown to be subserved by the dorsal striatum in rats and humans (Packard et al., 1989; Packard and McGaugh, 1996; Yin and Knowlton, 2006). When learning a new environment, both place learning and response learning strategies are implicated (Devan et al., 1999; Voermans et al., 2004; Miyoshi et al., 2012; Woolley et al., 2013; Ferbinteanu, 2016). However, it is often reported that during the early stages of navigation both rats and humans predominantly rely on hippocampal place learning, whereas response learning strategies become more prominent as the environment and specific routes become familiar, such as when the same route is taken repeatedly (Packard and McGaugh, 1996; Packard, 1999; Chang and Gold, 2003b; Hartley et al., 2003; Iaria et al., 2003; Poldrack and Packard, 2003; Schmitzer-Torbert, 2007; Jacobson et al., 2012).

As discussed above, the ability of individuals with DS to learn a route in a virtual environment was reported as relatively preserved. Individuals with DS were able to learn the routes and did not need significantly more trials than MA-matched TD children to reach criterion (Courbois et al., 2013). Given the very short routes to be learned in that study (only two turns for each route; right, left for the first route; left, right for the second route), individuals with DS may have learned a sequence of turns along the routes, while paying very little attention to landmarks around the routes and without integrating the two routes into an allocentric spatial frame of reference (see place learning, above). To our knowledge, only one study specifically assessed response learning in individuals with DS in a real-world environment (Mangan, 1992). The experimental setup consisted of a round platform (3.65 m in diameter) containing 11 symmetrically arranged holes that could hide rewards. The response learning task required 16–28 months old individuals with DS and TD children to always turn in the same direction on the platform to find the reward. For example, after watching the reward being hidden in one of the four holes surrounding the center hole (and always the same hole for any given child), the child was moved to the center of the platform. From here, if s/he turned to the right, for example, s/he would always find the rewarded hole. Although children with DS needed more trials than TD children to solve the task, they were able to find the reward in a final probe trial, following a 1-min delay between when the object was hidden and when the child was allowed to search. These results suggested that individuals with DS are capable of response learning from 16 months of age. Note that the same participants were tested on a place learning task, but consistent with the results of other studies describing the emergence of place learning between 21 and 25 months of age (Newcombe et al., 1998; Ribordy Lambert et al., 2013; Ribordy Lambert et al., 2015), neither DS nor TD children were able to exhibit reliable place learning. The inability of individuals with DS to exhibit place learning was thus inconclusive.

### Parallel Spatial Learning Systems

Even though “hippocampus-dependent” place learning and “striatum-dependent” response learning both contribute to spatial navigation, it has been hypothesized that the interaction between the two systems is competitive, rather than cooperative, and that the use or activation of one system inhibits the use or activation of the other system (Schroeder et al., 2002; White and McDonald, 2002; Chang and Gold, 2003b; Jacobson et al., 2012; Packard and Goodman, 2013). Importantly, studies in rats have demonstrated that response learning is dominant and even facilitated (i.e., more easily expressed) when the hippocampus is inactivated. By contrast, place learning is dominant when the striatum is inactivated (Packard and McGaugh, 1996; Schroeder et al., 2002; Chang and Gold, 2003b).

In that context, Bostelmann et al. (2017) tested individuals with Williams syndrome (WS), a genetic disorder associated with hippocampal abnormalities (Meyer-Lindenberg et al., 2005, 2006; Haas et al., 2014), using two basic spatial memory tasks designed to resemble the work carried out in rodents (Schroeder et al., 2002; White and McDonald, 2002; Chang and Gold, 2003b; Jacobson et al., 2012; Packard and Goodman, 2013). In the place learning task, individuals with WS exhibited severe impairments in comparison to MA-matched TD children. By contrast, in the response learning task, individuals with WS exhibited better performance than MA-matched TD. In the context of the above-described results from the lesion studies carried out in rats (Packard and McGaugh, 1996; Schroeder et al., 2002; Chang and Gold, 2003b), the performance of individuals with WS suggested that the impairment of the “hippocampus-dependent” place learning system is accompanied by the facilitation of the “striatum-dependent” response learning system in WS. It thus stands to reason that impaired hippocampal function in individuals with DS, as evidenced by deficits in high-resolution hippocampus-dependent spatial learning (Banta Lavenex et al., 2015), may result in a facilitation of response learning.

### Aim of the Study

The aim of the current study was to assess the capacity of individuals with DS to perform low-resolution spatial learning, based on two dissociable memory systems: (1) the place learning system, which depends on the hippocampus and creates flexible relational representations of the environment; and (2) the response learning system, which depends on the striatum and creates fixed stimulus–response representations of behavioral actions.

We hypothesized that the majority of individuals with DS should exhibit performance similar to that of MA-matched TD children and thus to succeed at a low-resolution place learning task. However, since aspects of their place learning abilities are nevertheless impaired, suggesting hippocampal dysfunction, individuals with DS should perform better than MA-matched TD children in a low-resolution response learning task.

## Materials and Methods

### Participants

Participants were 27 individuals with DS (13 males, 14 females), 19 typically developing children (10 males, 9 females), and 21 typically developed young adults (10 males, 11 females) (Table 1). Individuals with DS were recruited in Switzerland (*n* = 5) and in Italy (*n* = 22). All TD children and TD adults were recruited in Switzerland. Since no behavioral or performance differences were observed between the Swiss and the Italian participants with DS the data for these two populations were grouped for analysis and presentation. The parents and/or caregivers of participants with DS were asked whether the individual exhibited any possible signs of age-related dementia. None of our participants was signaled as showing any signs of dementia onset.

**Table 1:** Participants.

	Chronological age (years)				Mental age (years)			
		
	Mean	SD	Min	Max	Mean	SD	Min	Max
DS	23.4	7.7	15.0	43.5	5.6	0.7	4.7	7.0
TD-C	5.5	1.2	3.5	8.1	6.6	1.3	4.7	8.7
TD-A	21.0	2.0	18.3	25.5	–	–	–	–


*Chronological and mental ages for individuals with DS (*n* = 27; *NB*: the MA for three individuals with DS was not available), typically developing children (TD-C, *n* = 19), and typically developed young adults (TD-A, *n* = 21).*

Typically developing children with a chronological age similar to the MAs of the individuals with DS were recruited and tested in Switzerland. The MA of all TD children and 24 of 27 participants with DS was determined using the Leiter International Performance Scale-Revised (Leiter-R; Subtests included in the Brief IQ from which MA is calculated are: Figure Ground, Form Completion, Sequential Order, and Repeated Patterns) (Roid and Miller, 1997). TD children were reported by their parents to have been typically developing, and were neither born prematurely, nor had any suspected or diagnosed neurological conditions or learning disabilities. Two TD children (siblings) had strabismus that had been corrected, but both were diagnosed as lacking stereoscopic vision (i.e., depth perception). Since these two children behaved in a typical manner for the TD children, we included them in the study. The statistical analyses led to the same conclusions whether we included these two children or not. In sum, we did not exclude any recruited individuals with DS or TD children from our study. The TD adult group was not specifically matched for chronological age with our DS participants, although the mean age of the two groups did not differ statistically. TD adults reported to have been typically developing, and were neither born prematurely, nor had any suspected or diagnosed neurological conditions or learning disabilities. The results of the TD children were previously published in Bostelmann et al., 2017.

Participants were tested on the place learning task and the response learning task anywhere from 1 day to several months apart. Each session lasted approximately 45 min. Testing took place between 8:00 a.m. and 6:30 p.m. Human subjects research was approved by the Cantonal Ethics Commission for Human Research (Vaud, Switzerland; protocol no. 60/14), and was in accordance with the code of ethics of the World Medical Association (Declaration of Helsinki) for experiments involving human subjects in research. The participants (TD adults and some individuals with DS) or the parents of the TD children and the participants with DS gave informed written consent.

### Testing Facilities

**FIGURE 1:**
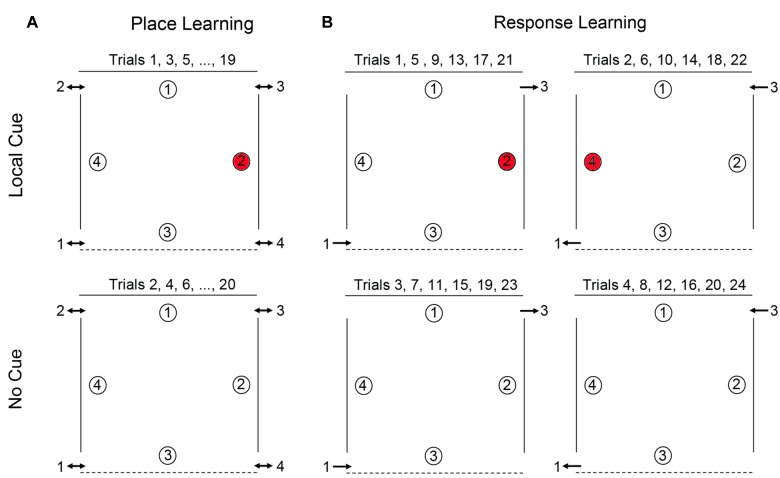
Schematic representation of the open-field arena used for testing place learning **(A)** and response learning **(B)**. For place learning, the reward was always placed at the same location within the arena (location 2 for 50% of the participants and location 4 for the other 50% of the participants). For response learning, the reward (at either location 2 or location 4 on alternate trials) could be found by performing the same motor response upon entering the arena.

Five individuals with DS, and all the TD children and TD adults were tested at the University of Lausanne, Switzerland. Twenty-two individuals with DS were tested in Nardò, Italy. The main features of the testing facilities were consistent between the two sites. Testing took place within large rectangular rooms (8 m × 8 m in Vaud and 16 m × 10 m in Nardò) containing many polarizing features such as doors, tables, chairs, wall posters, etc. Within the room was a 4 m × 4 m testing arena (Figure 1) that consisted of three walls made of suspended, opaque curtains (2 m high). Whereas the curtain on the back wall was 4 m wide, the curtains on the side walls extended only 3 m, so that there was a 50 cm gap at the front and the back of the wall, thus creating four entry points (“doors”) through which participants passed in order to enter and exit the arena. The fourth (front) boundary of the arena was delineated by a rope extending to the two opposing sides of the arena, and suspended 30 cm off the ground. Exterior to the two side walls, the inter-trial waiting area was a corridor (2 m × 4 m) that contained two chairs with their backs to the arena and objects that were unique to each side. From within the arena, and from the inter-trial waiting area, participants had access to distant visual cues in front of the arena. Objects found in front of the arena (a table, chairs, one experimenter, camera, etc.) were placed 2 m away from the front of the arena.

The arena’s floor was uniform and thus provided no visual guidance cues. The testing arena was empty except for four white paper plates (18 cm in diameter) placed at the cardinal points in the arena (Figure 1). An inverted opaque plastic cup (7.5 cm in diameter, 6.5 cm high) was placed on each paper plate. A reward was placed under the inverted cup at one location. Participants had to lift or turn over the plastic cups to obtain the reward. Rewards were coins for individuals with DS and TD adults, and “treats” (e.g., Smarties^®^, Goldfish^®^ crackers, pieces of breakfast cereal or pretzels) for TD children. TD adults and parents of TD children were queried with respect to alimentary allergies prior to testing, and children were asked whether there were any treats that they did not like, which would be excluded as rewards during their testing session. All testing was videotaped with a video camera located in front of the arena.

### General Testing Procedures

For individuals with DS and TD children, testing involved a team of two experimenters. Experimenter 1 (E1) would stay with the participant throughout the testing session and would enter the arena with the participant, encourage the participant to search for the hidden rewards, verbally praise the participant when a reward was found, remove cups from unrewarded locations as soon as they had been searched by a participant, direct the participant to the correct exit at the end of the trial, and occupy the participant during the inter-trial interval by reading to or conversing with the participant. Experimenter 2 (E2) was responsible for replacing the reward between trials, recording the data, and announcing the correct entry and exit doors to E1. Before testing began, participants viewed the arena with the four arranged plates (no inverted cups were present), from in front of the arena. While still in front of the arena, E1 showed the participant a reward item on a paper plate that she held in her hand. While the participant was watching, E1 would lower a plastic white cup over the reward to hide it. The participant would then be asked “Where is the treat/coin? Can you show me where it is?” When the participant lifted the cup to expose the reward, s/he would be verbally praised and told that it was hers/his to keep. Once the participant had been shown that a reward could be found underneath the plastic cup, the participant and E1 would go to the predetermined side of the arena where testing would begin. Once the participant was behind the curtain and occupied, E2 would hide a reward at the predetermined reward location. For TD adults, only experimenter E2 was present, and the participants were directed to the correct entrances when E2 called out a number that hung next to the entrance on the outside of the arena, or when E2 pointed to the specific exit when they were inside the arena.

In both the place and response learning tasks, participants completed two different types of trials: (1) *Local cue trials*, in which a local cue, specifically a red cup, covered the reward, whereas the three non-rewarded locations were covered with white cups (Figure 1). To find the reward, participants could search for the red cup or rely on place or response learning to identify the reward location (see below). This condition allowed us to assess the participants’ motivation to search for the reward and their overall understanding of the task. (2) *No cue trials*, in which no local cue marked the reward location, as identical white cups covered all locations. In this case, participants could not discriminate between rewarded and never-rewarded locations based on local features, but instead had to rely on either a place learning strategy or a response learning strategy to identify the rewarded location. Half of the participants performed the place learning task first, the other half performed the response learning task first. Analyses showed that performance was not influenced by whether participants completed the place learning task or the response learning task first.

### Place Learning

Place learning was assessed by testing the ability of individuals with DS, TD children, and TD adults to learn and remember the location of one reward among four potentially rewarded locations (Figure 1A). For each participant, one location in the arena was chosen as the goal location: for half of the participants, location 4 was the designated goal, for the other half location 2 (locations 1 and 3, whose positions were distinct, were never goal locations). Each participant completed a total of 20 alternating local cue and no cue trials, with a 15-min break after the first 10 trials (*NB*: TD adults did not receive a break). There were four entries and exits to the arena. In order to preclude the use of egocentric and response strategies, participants were obliged to enter and exit the arena from a different door for every trial, and could never enter through a door they had just exited through on the immediately preceding trial. Entry order was determined in a pseudo-random manner, with respect to the following conditions: (1) all entrances should be used an equal number of times in the two conditions (local and no cue conditions); (2) participants may never enter the arena through a door which they had just exited on the immediately preceding trial (to preclude the use of egocentric strategies); (3) a no cue trial may never have the same entrance as the immediately preceding local cue trial; and (4) all entries must be made from the same side (right or left) that the participant just exited on the previous trial (i.e., participants were not moved from one side of the arena to the other between trials). At the end of the trial, E2 would point to the appropriate exit and E1 would guide the participant to that exit by pointing or by heading there first, therefore, ensuring that the participant was on the appropriate side of the arena for the next trial. Participants were thus constantly moving about the arena from trial to trial, entering and exiting on different sides, and at the back or front of the arena. Moreover, no environmental landmarks, such as doors, windows, or furniture, could be found adjacent to or directly behind any of the reward locations (with the exception of the red cups in the local cue condition). Consequently, in order to identify the reward location in the absence of the local cue, participants must rely on place learning: they must be able to learn and remember the reward location in relation to distal objects in the environment.

### Response Learning

Response learning was assessed by testing the ability of individuals with DS, TD children, and TD adults to learn and remember the location of one reward. In this task, location 2 and location 4 were alternately rewarded. On all odd numbered trials participants entered through door 1, location 2 was rewarded, and exited through door 3 (Figure 1B). On all even-numbered trials, participants entered through door 3, location 4 was rewarded, and exited through door 1. Participants thus had to learn that they could find the reward by performing a fixed motor response from the entrance point. Each participant completed pairs of local cue trials and pairs of no cue trials in alternation (2 local cue trials followed by 2 no cue trials for a total of 24 trials total), with a 15-min break after the first 12 trials. Response learning proceeded as follows: Trials 1, 5, 9, 13, 17, and 21, with Local Cue: enter door 1, location 2 rewarded, exit door 3. Trials 2, 6, 10, 14, 18, and 22, with Local Cue: enter door 3, location 4 rewarded, exit door 1. Trials 3, 7, 11, 15, 19, and 23, No Cue: enter door 1, location 2 rewarded, exit door 3. Trials 4, 8, 12, 16, 20, and 24, No Cue: enter door 3, location 4 rewarded, exit door 1. Thus, from trial-to-trial, the location of the reward changed in relation to the global environment, but remained constant relative to the door just used by the participant to enter the arena. Consequently, in order to identify the reward location in the absence of the local cue, participants must rely on response learning: they must be able to learn to associate the reward location with a fixed motor response from the door used to enter the arena.

### Verbal Instructions and Feedback

For the first local cue trial of both the place and response learning tasks, as participants first entered the arena, E1 would ask the participant “Where do you think the reward is hidden?”. For each subsequent trial, upon entering the arena, E1 would simply prompt the participant by saying “Show me where the reward is hidden”. In order to determine unequivocally whether individuals with DS could succeed at place learning when given access to coherent visual, vestibular and proprioceptive information, and to preclude poor task comprehension from negatively influencing performance, after individuals with DS, TD children, and TD adults found the reward on the first two trials (one local cue trial and one no cue trial) of the place learning task, E1 explained that the reward would always be found at this exact same location (while pointing at the rewarded plate with the red cup hidden from view). This same explanation was repeated to individuals with DS that did not identify the rewarded location for up to the first five local cue and no cue trials (or until they became annoyed, told E1 that they remembered the rule, and asked E1 to stop repeating that instruction). For the response learning task, the experimenter gave no explanation to the participants about finding the reward in any particular location since the premise behind response learning is that individuals rely on a stimulus–response “habit” (“I do this”), and thus the rule is the solution. Note that for the place learning task, the rule (“always here”) is not the same as the solution (i.e., using place learning to identify where “here” is). Thus, although participants were told the rule, they would not be able to follow this rule if they were not capable of place learning (i.e., identifying the target location relative to distal objects and locations in the environment).

### Data Analysis

We performed general linear model (GLM) analyses to compare the number of correct first choices (i.e., choosing the reward location as their first choice upon entering the arena) that participants made on the last eight trials (thus omitting the first two local cue trials and the first two no cue trials in the place learning task, and the first two pairs of trials – the first two pairs of local cue trials and the first two pairs of no cue trials – in the response learning task), between groups (DS individuals, TD children, and TD adults), between conditions (local cue vs. no cue), and between spatial tasks (place learning vs. response learning). All statistical analyses were performed with the SPSS 21.0 software. *Post hoc* analyses were performed with the Fisher-least significant difference test when the ANOVA *F* ratio was significant, thus controlling for Type I error rate (Carmer and Swanson, 1973). Significance level was set at *P* < 0.05 for all analyses. We report effect size with $\eta_{p}^{2}$ [partial eta squared: SSeffect/(SSeffect + SStotal); the sum of squares of the effect divided by the total sum of squares + the sum of squares of the effect; as reported by SPSS 21.0] for ANOVAs, as well as Cohen’s ds [difference between means/pooled standard deviation; or ds = *t*^∗^sqrt(1/*n*1 + 1/*n*2)] for unpaired t-tests and Cohen’s *dz* (*dz* = *t*/ sqrt(*n*)) for paired samples *t*-tests (Lakens, 2013).

The data on the number of correct choices suggested that individuals with DS and TD children exhibited a bimodal performance distribution. The majority of individuals with DS made very few or no errors on the place learning task, whereas a number of individuals made very few or no correct choices. This suggests that the average number of correct choices alone may not be sufficient to represent task performance. To provide additional information, we compared the number of individuals who performed above chance on each task across the three groups. Above chance performance in the place and response learning tasks was determined for each individual with a non-parametric Wilcoxon signed-rank test comparing the number of correct first choices (visiting a rewarded location) and the number of incorrect first choices (visiting a non-rewarded location) for the last eight place and response learning trials. Since an analysis of choices for all individuals, both those that passed and those that did not pass, showed that errors were distributed across the three incorrect locations (and were not restricted to the opposite location), we normalized the number of correct and incorrect choices based on the probability to make those choices: the number of correct choices was divided by one and the number of incorrect choices was divided by three. Importantly, when calculated as such, this level of above chance performance is equivalent to making six correct first choices on the last eight trails (i.e., 75% correct). The number of individuals with DS, MA-matched TD children, and TD adults who solved one, two, or neither of these tasks were compared with the log-likelihood ratio for contingency tables (Zar, 1999).

## Results

**FIGURE 2:**
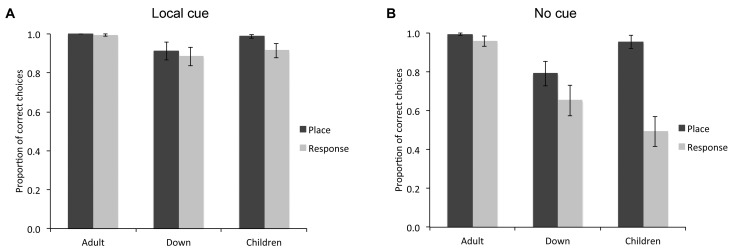
Proportion of correct choices (i.e., choice of the rewarded location as the first choice upon entering the arena; mean ± SEM) made by individuals with DS (*n* = 27), TD children (*n* = 19), and TD adults (*n* = 21) in the place learning and response learning tasks, in presence **(A)** or absence **(B)** of a local cue marking the reward location.

In order to characterize the place learning and response learning capacities of individuals with DS, MA-matched TD children, and TD adults, we analyzed the proportion of correct choices (i.e., choosing the reward location as their first choice upon entering the arena) in both the place learning and response learning tasks, in presence or absence of a local cue (a red cup) marking the reward location (Figure 2). A global GLM analysis revealed differences between experimental groups [*F*_(2,64)_ = 8.460, *P* = 0.001, $\eta_{p}^{2}$ = 0.209 = 0.209, power = 0.958; TD adults > TD children = DS individuals] and cue conditions [*F*_(1,64)_ = 33.199, *P* < 0.001, $\eta_{p}^{2}$ = 0.342, power = 1.000; Local cue > No cue], as well as an interaction between groups and cue conditions [*F*_(2,64)_ = 6.058, *P* = 0.004, $\eta_{p}^{2}$ = 0.159, power = 0.870]. There was also a difference between the two types of spatial tasks [place learning vs response learning: *F*_(1,64)_ = 18.680, *P* < 0.001, $\eta_{p}^{2}$ = 0.226, power = 0.989]. Moreover, there were significant interactions between cue conditions and spatial tasks [*F*_(1,64)_ = 21.334, *P* < 0.001, $\eta_{p}^{2}$ = 0.250, power = 0.995], between spatial tasks and experimental groups [*F*_(2,64)_ = 6.050, *P* = 0.004, $\eta_{p}^{2}$ = 0.159, power = 0.870], and between cue conditions, spatial tasks, and experimental groups [*F*_(2,64)_ = 7.306, *P* = 0.001, $\eta_{p}^{2}$ = 0.186, power = 0.927].

### Local Cue

When a red cup marked the goal location (Figure 2A), the difference between groups just failed to reach significance [*F*_(2,64)_ = 3.046, *P* = 0.054, $\eta_{p}^{2}$ = 0.087, power = 0.570], although individuals with DS (*M* = 0.899, *SE* = 0.026) made fewer correct choices than TD adults (*M* = 0.997, *SE* = 0.030; *P* = 0.017). There was no difference in performance between place learning and response learning tasks [*F*_(1,64)_ = 2.314, *P* = 0.133, $\eta_{p}^{2}$ = 0.035, power = 0.322] and no interaction between spatial tasks and experimental groups [*F*_(2,64)_ = 0.653, *P* = 0.524, $\eta_{p}^{2}$ = 0.020, power = 0.155]. In the place learning task with a local cue, there was no difference between groups [*F*_(2,64)_ = 2.356, *P* = 0.103, $\eta_{p}^{2}$ = 0.069, power = 0.460]. In the response learning task with a local cue, there was no significant difference between groups [*F*_(2,64)_ = 2.246, *P* = 0.114; $\eta_{p}^{2}$ = 0.066, power = 0.441].

Within group comparisons revealed that TD children made more correct choices in the place learning task than in the response learning task [*t*_(18)_ = 2.158, *P* = 0.045, Cohen’s *dz* = 0.495; place learning: *M* = 0.987, *SE* = 0.009; response learning: *M* = 0.915, *SE* = 0.037). The performance of TD adults [*t*_(20)_ = 1.000, *P* = 0.329, Cohen’s *dz* = 0.218) and individuals with DS [*t*_(26)_ = 0.536, *P* = 0.596, Cohen’s *dz* = 0.103] did not differ between the place learning and response learning tasks.

In sum, in the presence of a red cup marking the reward location, although TD adults, TD children and individuals with DS performed well above chance, TD children made more correct choices in the place learning task than in the response learning task, whereas no difference was found between the two tasks for the DS group, or the TD adult group.

### No Cue

When the reward location was not marked by a local cue (Figure 2B), there was a difference between experimental groups [*F*_(2,64)_ = 9.619, *P* < 0.001, $\eta_{p}^{2}$ = 0.231, power = 0.977], a difference between place learning and response learning tasks [*F*_(1,64)_ = 24.492, *P* < 0.001, $\eta_{p}^{2}$ = 0.277, power = 0.998], and an interaction between spatial learning tasks and experimental groups [*F*_(2,64)_ = 8.119, *P* = 0.001, $\eta_{p}^{2}$ = 0.202, power = 0.951]. In the place learning task without a local cue, there was a difference between groups [*F*_(2,64)_ = 5.489, *P* = 0.006, $\eta_{p}^{2}$ = 0.146, power = 0.834]. TD adults (*M* = 0.994, *SE* = 0.049) and TD children (*M* = 0.955, *SE* = 0.051) performed similarly (*P* = 0.579), and made more correct choices than individuals with DS (*M* = 0.793, *SE* = 0.043; both *P* < 0.05). Note that if we consider only the performance of the individuals performing above chance level (including 21/21 TD adults, 21/27 individuals with DS, and 18/19 TD children), the overall statistical difference between groups remained [*F*_(2,57)_ = 3.186, *P* = 0.049, $\eta_{p}^{2}$ = 0.101, power = 0.587]. Specifically, individuals with DS (*M* = 0.953, *SE* = 0.018) made fewer correct choices than TD adults (*M* = 0.994, *SE* = 0.006; *P* = 0.02), but their performance did not differ from that of TD children (*M* = 0.987, *SE* = 0.009; *P* = 0.067).

In the response learning task without a local cue, there was a difference between groups [*F*_(2,64)_ = 10.730, *P* < 0.001, $\eta_{p}^{2}$ = 0.251, power = 0.987]. TD adults (*M* = 0.959, *SE* = 0.071) performed overall better than individuals with DS (*M* = 0.654, *SE* = 0.062) and TD children (*M* = 0.495, *SE* = 0.074; both *P* < 0.01), but individuals with DS performed similarly to TD children (*P* = 0.173). Note that if we consider only the performance of the individuals performing above chance level (including 17/21 TD adults, 15/27 individuals with DS, and only 3/19 TD children), there was no difference between groups [*F*_(2,32)_ = 0.419, *P* = 0.662, $\eta_{p}^{2}$ = 0.025, power = 0.112; DS individuals: *M* = 0.992, *SE* = 0.008; TD adults: *M* = 0.978, *SE* = 0.016; TD children: *M* = 1.000, *SE* = 0.000].

The performance of TD adults did not differ between place learning and response learning tasks [*t*_(20)_ = 1.305, *P* = 0.207, Cohen’s *dz* = 0.284; place learning: *M* = 0.994, *SE* = 0.006; response learning: *M* = 0.959, *SE* = 0.026]. Similarly, the performance of individuals with DS did not differ between the two learning tasks [*t*_(26)_ = 1.650, *P* = 0.111, Cohen’s *dz* = 0.317; place learning: *M* = 0.793, *SE* = 0.063; response learning: *M* = 0.654, *SE* = 0.079]. By contrast, TD children made more correct choices in the place learning task than in the response learning task [*t*_(18)_ = 5.561, *P* < 0.001, Cohen’s *dz* = 1.275; place learning: *M* = 0.955, *SE* = 0.033; response learning: *M* = 0.495, *SE* = 0.078].

In sum, as a group, in the place learning task without a local cue, individuals with DS made fewer correct choices than TD children and TD adults. In the response learning task without a local cue, individuals with DS made fewer correct choices than TD adults, but performed similarly to TD children. TD children performed better in the place learning task than in the response learning task, whereas individuals with DS performed similarly in both tasks.

### Within Group Comparisons Across All Conditions

Within group comparisons of performance in the four experimental conditions confirmed the contrasting behavioral patterns observed for individuals with DS, TD children, and TD adults, in the place learning and response learning tasks.

The performance of TD adults did not differ between the four experimental conditions [*F*_(3,60)_ = 1.864, *P* = 0.145, $\eta_{p}^{2}$ = 0.085, power = 0.460].

Typically developing children made more correct choices when the local cue was present in the place learning task than in the response learning task, and more correct choices in both of these conditions than in the response learning task without the local cue [*F*_(3,54)_ = 28.748, *P* < 0.001, $\eta_{p}^{2}$ = 0.615, power = 1.000; place learning with local cue >response learning with local cue > response learning without local cue; all *P* < 0.05]. In addition, without the local cue, TD children made more correct choices in the place learning task than in the response learning task (*P* < 0.001). By contrast, TD children did not differ in the number of correct choices in the place learning task with and without the local cue (*P* = 0.263).

The performance of individuals with DS also differed between testing conditions [*F*_(3,78)_ = 5.393, *P* = 0.002, $\eta_{p}^{2}$ = 0.172, power = 0.924]. Individuals with DS made more correct choices in the place learning task with the local cue than in the place learning and response learning tasks without the cue (both *P* < 0.05). In addition, individuals with DS made more correct choices in the response learning task with the cue than in the response learning task without the cue (*P* = 0.003).

In sum, contrary to TD children and TD adults, individuals with DS made fewer correct choices in the place learning task without the local cue than with the local cue. In the response learning task, TD children and individuals with DS made fewer correct choices in the absence of the local cue than when the local cue marked the reward location.

### Individual Analyses

**FIGURE 3:**
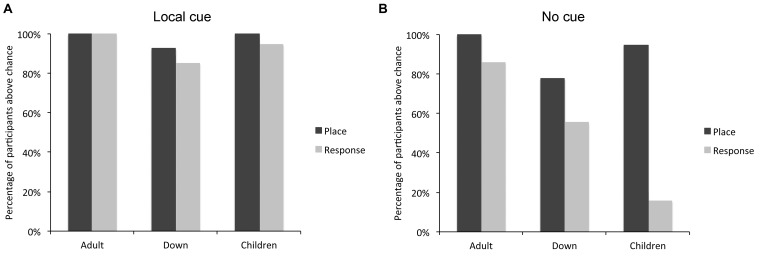
Percentage of individuals with DS (*n* = 27), TD children (*n* = 19), and TD adults (*n* = 21) who performed above chance level in the place learning and response learning tasks, in presence **(A)** or absence **(B)** of a local cue marking the reward location.

We also determined how many individuals with DS, TD children, and TD adults performed above chance level, and therefore demonstrated their ability to solve either the place learning or response learning tasks, in presence or absence of the local cue (Figure 3). In the presence of the local cue, the number of participants who performed above chance level in the place learning and response learning tasks did not differ between groups [log likelihood ratio test: *X*^2^_(2)_ = 0.040, *P* = 0.980]. Moreover, there was no difference in the number of participants who successfully performed the place learning or response learning task within each group [TD adults: *X*^2^_(1)_ = 0, *P* = 1; individuals with DS: *X*^2^_(1)_ = 0.763, *P* = 0.382; TD children: *X*^2^_(1)_ = 1.413, *P* = 0.234].

By contrast, in absence of the local cue, the number of participants who succeeded (i.e., performed above chance level) at the place learning and response learning tasks differed between groups [log likelihood ratio test: *X*^2^_(2)_ = 7.059, *P* = 0.029]. The number of individuals with DS who succeeded at the place learning and response learning tasks did not differ [*X*^2^_(1)_ = 3.043, *P* = 0.081]. By contrast, more TD children succeeded at the place learning task than the response learning task [*X*^2^_(1)_ = 27.848, *P* < 0.0001], and more TD adults succeeded at the place learning task than the response learning task [*X*^2^_(1)_ = 4.390, *P* = 0.037]. For TD adults, the difference was due to the fact that whereas all TD adults succeeded at place learning, three reported using a conditional place strategy to solve the response task (i.e., they remembered which location was rewarded last and chose the opposite location) and one simply failed to identify the rewarded locations consistently. The other 17 TD adults both succeeded at the task and reported using a response strategy. By definition, the use of a response strategy implies that the participant does not use a cognitive place strategy to identify the rewarded location; instead, they recall only the rule “when I am here, I go there.” In sum, whereas more TD children (and TD adults) succeeded at the place learning task compared to the response learning task, this difference was not significant in DS.

In addition, we further determined how many individuals with DS, TD children, and TD adults demonstrated their ability to solve both, either or none of the place learning or response learning tasks when no local cue marked the reward location, in order to assess whether the ability to complete one task may be associated with the inability to complete the other task (Table 2).

**Table 2:** Individual performance.

	Place Yes	Place Yes	Place No	Place No
	Response Yes	Response No	Response Yes	Response No
DS individuals	13	8	2	4
TD children	3	15	0	1
TD adults	17	4	0	0


*Number of individuals with DS (from *n* = 27), TD children (from *n* = 19), and TD adults (from *n* = 21), who performed above chance level in the place learning and/or the response learning tasks in absence of the local cue.*

The number of participants who solved both, either or none of the two tasks differed between groups [log likelihood ratio test: *X*^2^_(6)_ = 27.159, *P* < 0.001]. In the place learning task, the number of participants who performed above chance level differed between groups [*X*^2^_(2)_ = 8.425, *P* = 0.015]. Fewer individuals with DS performed above chance level than TD adults [*X*^2^_(1)_ = 7.566, *P* = 0.006]. TD children did not differ from TD adults [*X*^2^_(1)_ = 1.517, *P* = 0.218] or individuals with DS [*X*^2^_(1)_ = 2.795, *P* = 0.094]. In the response learning task, the number of participants who performed above chance level differed between groups [*X*^2^_(2)_ = 18.627, *P* < 0.001]. Fewer TD children performed above chance level than TD adults [*X*^2^_(1)_ = 18.427, *P* < 0.001] and individuals with DS [*X*^2^_(1)_ = 7.908, *P* < 0.005]; the difference between individuals with DS and TD adults failed to reach significance [*X*^2^_(1)_ = 3.559, *P* = 0.059].

In sum, the analyses of individual performance suggest that TD adults can succeed at both place and response learning, whereas TD children are preferentially place learners and have difficulty with response learning. By contrast, individuals with DS exhibit an intermediate pattern, with place learning capacities similar to those of TD children, but response learning capacities similar to those of TD adults.

### Choice Analyses

**FIGURE 4:**
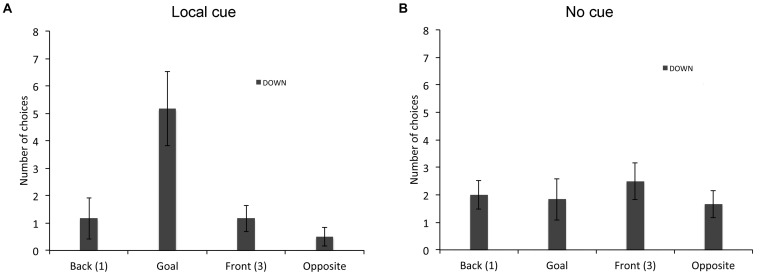
Proportion of choices (goal location (2), opposite location (4), back location (1), or front location (3), as the first choice upon entering the arena; mean +/- SEM) made by individuals with DS, who did not perform above chance level the place learning task, in presence **(A)** or absence **(B)** of a local cue marking the reward location.

Finally, we analyzed the types of choices made by individuals who did not perform above chance level in the place or response learning task in absence of the local cue. In the place learning task, since all TD adults performed the task and only one of the 19 TD children did not succeed without the local cue, we restricted this analysis to the six individuals with DS who did not perform above chance level in absence of the local cue (Figure 4). A repeated-measures GLM analysis revealed no main effect of locations [*F*_(3,15)_ = 2.283, *P* = 0.121, $\eta_{p}^{2}$ = 0.313, power = 0.465], but a significant interaction between locations and cue conditions [*F*_(3,15)_ = 5.445 *P* = 0.010, $\eta_{p}^{2}$ = 0.521, power = 0.857]. In presence of the local cue (Figure 4A), the six individuals with DS chose preferentially the goal location over the opposite location [*F*_(3,15)_ = 5.000, *P* = 0.013, $\eta_{p}^{2}$ = 0.500, power = 0.824; goal > opposite: *P* = 0.031]. By contrast, in absence of the local cue (Figure 4B), individuals with DS did not discriminate between the four locations [*F*_(3,15)_ = 0.255, *P* = 0.856, $\eta_{p}^{2}$ = 0.049, power = 0.088], suggesting that as a group they exhibited no consistent pattern of behavior (e.g., a response strategy would result in the “opposite” location being chosen on 50% of the trials, whereas a “first cup seen” strategy would result in locations 1 (“Back”) and 3 (“Front”) each being chosen 50% of the time).

**FIGURE 5:**
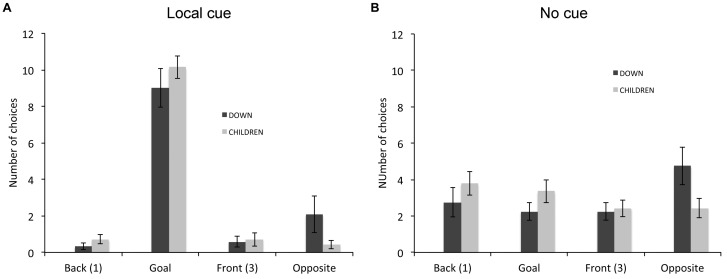
Proportion of choices (goal location (2), opposite location (4), back location (1), or front location (3), as the first choice upon entering the arena; mean ± SEM) made by individuals with DS and TD children, who did not perform above chance level the response learning task, in presence **(A)** or absence **(B)** of a local cue marking the reward location.

In the response learning task (Figure 5), we compared the choices of individuals with DS (*n* = 12) and TD children (*n* = 16) that did not perform above chance. A repeated-measures GLM analysis revealed a main effect of locations [*F*_(3,72)_ = 27.999, *P* < 0.001, $\eta_{p}^{2}$ = 0.538, power = 1.000], an interaction between groups and locations [*F*_(3,72)_ = 2.876, *P* = 0.042, $\eta_{p}^{2}$ = 0.107, power = 0.664] and an interaction between conditions and locations [*F*_(3,72)_ = 60.626, *P* < 0.001, $\eta_{p}^{2}$ = 0.716, power = 1.000]. The analysis of the choices of individuals with DS revealed a main effect of locations [*F*_(3,33)_ = 7.639, *P* = 0.001, $\eta_{p}^{2}$ = 0.410, power = 0.977], and an interaction between locations and cue conditions [*F*_(3,33)_ = 22.822 *P* < 0.001, $\eta_{p}^{2}$ = 0.675, power = 1.000]. In presence of the local cue (Figure 5A), individuals with DS chose preferentially the goal location over the other three locations [*F*_(3,13)_ = 22.317, *P* < 0.001, $\eta_{p}^{2}$ = 0.670, power = 1.000; all *P* < 0.01). By contrast, in absence of the local cue (Figure 5B), individuals with DS did not discriminate between the four locations [*F*_(3,33)_ = 1.955, *P* = 0.140, $\eta_{p}^{2}$ = 0.151, power = 0.458]. The analysis of the choice of TD children also revealed a main effect of locations [*F*_(3,39)_ = 32.364, *P* < 0.001, $\eta_{p}^{2}$ = 0.713, power = 1.000], and an interaction between locations and cue conditions [*F*_(3,39)_ = 41.139, *P* < 0.001, $\eta_{p}^{2}$ = 0.760, power = 1.000]. In presence of the local cue (Figure 5A), TD children chose preferentially the goal location over the other three locations, which did not differ from each other [*F*_(3,39)_ = 109.792, *P* < 0.001, $\eta_{p}^{2}$ = 0.894, power = 1.000; all *P* < 0.001]. By contrast, in absence of the local cue (Figure 5B), TD children did not discriminate between the four locations [*F*_(3,39)_ = 1.058, *P* = 0.378, $\eta_{p}^{2}$ = 0.075, power = 0.264].

*In sum*, in the place learning task, although the average number of correct choices made by individuals with DS was lower than those made by MA-matched TD children and TD young adults, the number of individuals with DS who performed above chance level did not differ from TD children, suggesting a relative preservation of low-resolution place learning abilities in DS. In the response learning task, the average performance of individuals with DS was lower than that of TD adults, but it did not differ from that of TD children. Moreover, the number of individuals with DS who performed the response learning task above chance level did not differ from TD adults, and was higher than that of TD children, suggesting a slight enhancement of low-resolution response learning abilities in DS.

## Discussion

We investigated the capacities of individuals with DS to solve low-resolution, place learning and response learning tasks, which are thought to be subserved by two different functional brain networks. Individuals with DS exhibited relatively preserved low-resolution place learning capacities and somewhat facilitated response learning capacities, as compared to TD children. Together with our previous findings of severe impairments in high-resolution place learning capacities in DS (Banta Lavenex et al., 2015), and our previous findings of severe impairments in low-resolution place learning and facilitated response learning in WS (Bostelmann et al., 2017), our current findings support the hypothesis that impairments in some components of the “hippocampus-dependent” place learning system may facilitate “striatum-dependent” response learning.

### Place Learning Capacities in DS

First, it is important to emphasize that all participants from the three different groups (individuals with DS, MA-matched TD children and TD adults) were able to discriminate the rewarded location in presence of the local cue, in both the place learning and response learning tasks. This finding shows that individuals with DS (1) understood the objectives of the task; (2) could initiate and sustain a selective search; and (3) inhibit searching unrewarded locations when they knew the location of the hidden reward. In the place learning task, when no local cue marked the reward location, as a group individuals with DS made fewer correct choices, as compared to both TD children and TD adults. However, since six of 27 individuals with DS made numerous errors and 21 individuals with DS performed above chance and made very few errors, treating all individuals with DS as a homogeneous group may not be most appropriate way to describe their capacities. Indeed, when considering only the individuals who performed above chance level, although individuals with DS made fewer correct choices than TD adults, their performance did not differ from that of TD children. Moreover, the average number of correct choices may not be the most appropriate indicator of group performance. By contrast, we found that the number of individuals with DS who performed the place learning task above chance level was not significantly different from TD children. Altogether, these findings suggest that although individuals with DS did not reach the performance level of TD adults, they exhibited a relative preservation of low-resolution place learning, with 21/27 of individuals with DS exhibiting capacities similar to those of MA-matched TD children.

By contrast, we have previously shown that individuals with DS were severely impaired, as compared to MA-matched TD children, in a high-resolution place learning task in which participants had to find three rewards among twelve potentially rewarded locations (Banta Lavenex et al., 2015). Indeed, only 50% of the individuals with DS in that study performed above chance level. Moreover, these individuals choose preferentially the rewarded location located on the outer array, which could be identified by using a low-resolution topological representation of the environment (Poucet and Benhamou, 1997). TD children, on the other hand, consistently showed that they were able to identify the other two rewarded locations located on the middle and inner arrays, which required the ability to build a high-resolution spatial representation of the environment. Only two individuals with DS (10%) were able to reliably identify the two rewarded locations on the middle and inner arrays. Our previous findings thus differ from those of the present study in which we found that 78% of the individuals with DS were capable of succeeding at a low-resolution place learning task.

We believe that the overall pattern of results exhibited by individuals with DS as compared to TD children and adults, rather than any single measure, is informative for deciphering the relative preservation or impairment of their spatial learning and memory capacities. The majority of individuals with DS demonstrate relatively preserved low-resolution place learning capacities (similar to TD children, but impaired as compared to TD adults), but severely impaired high-resolution place learning capacities (as compared to TD children). These behavioral findings support the hypothesis that some specific hippocampal circuits may be particularly impacted in DS. Indeed, different functional pathways within the hippocampal formation are thought to contribute to complementary but partially dissociable spatial coordinate systems (Rolls and Kesner, 2006). A direct projection from the entorhinal cortex to CA1 is thought to be able to subserve basic allocentric spatial processing (Brun et al., 2002; Lavenex and Banta Lavenex, 2013). By contrast, imaging studies in humans, neurophysiological studies in rats, and computational models, have established that the dentate gyrus, together with its connections to CA3, subserve a process known as pattern separation (Kesner, 2007; Bakker et al., 2008), which subserves the discrimination of spatial locations that are close to one another (Kesner, 2007; Morris et al., 2012). In accordance with this hypothesis, disrupting the CA3 input to CA1 results in decreased spatial tuning of CA1 place cells (Brun et al., 2002; Nakashiba et al., 2008), suggesting the necessity of the dentate gyrus to CA3 functional circuit for building high-resolution spatial representations, even though it is not required for building low-resolution spatial representations. The fact that individuals with DS have relatively preserved low-resolution place learning capacities, but impaired high-resolution place learning capacities suggest that the function of CA1 may be relatively preserved, whereas the function of the dentate gyrus/CA3 region may be more generally and severely impaired. Indeed, although structural MRI studies have reported smaller hippocampal volumes in children (Pinter et al., 2001) and adults (Raz et al., 1995; Aylward et al., 1999) with DS, neuropathological findings suggest possibly greater abnormalities in the dentate gyrus (Contestabile et al., 2010). Non-invasive functional studies, as well as detailed post-mortem neuropathological studies will be needed to provide additional evidence necessary to answer this question.

### Response Learning Capacities in DS

The second aim of our study was to assess low-resolution response learning capacities in DS. When no cue indicated the location of the reward, individuals with DS exhibited a response learning performance that was intermediate between those of MA-matched TD children and TD adults. As a group, in the response learning task without the local cue, individuals with DS made fewer correct choices than TD adults, but their performance was not significantly different from that of MA-matched TD children. However, as for place learning, the group of DS individuals exhibited a bimodal performance, again suggesting that the average number of correct responses may not be the most appropriate indicator of group performance. Accordingly, when we consider the individual performance, more individuals with DS performed above chance level (15/27; 56%) than TD children (3/19; 16%). As discussed previously (Bostelmann et al., 2017) response learning appears to be inhibited in 3.5- to 8-year-old TD children. Although response learning may emerge as early as 6 months of age, and earlier than place learning during both typical and atypical development (Acredolo, 1978; Cornell and Heth, 1979; Mangan, 1992; Newcombe et al., 1998; Ribordy Lambert et al., 2013), once TD children start exhibiting basic place learning capacities around 2 years of age (Newcombe et al., 1998; Ribordy Lambert et al., 2013), incidental spatial response learning, as tested under these experimental conditions, appears to become extremely difficult for TD children. By contrast, our data show that response learning appears to be more easily expressed in individuals with DS as compared to TD children with the same MA.

Finally, it is important to consider the impact of the different instructions given for the different tasks. Since individuals with DS were hypothesized to show greater deficits in hippocampal-dependent tasks such as place learning, in order to try to assure that any deficits that we observed were due to deficits in place learning alone, and not to other parasitic cognitive processes (e.g., lack of comprehension of the goals of the task), we gave as much instruction as possible. This means that for the place learning task participants were told that the reward “can always be found here.” However, if the hippocampus is impaired and unable to support allocentric spatial processing, this instruction alone would not enable the participant to find the rewarded location. By contrast, for the response learning task, telling the participant to “go this way” would be providing the solution to the problem. Importantly, our results show that for response learning task, even given the more apparent difficulty of the task due to the lack of verbal instructions: (1) more individuals with DS passed than did TD children; and (2) the number of individuals with DS passing the place learning task did not differ from the number of DS individuals passing the response learning task. Together, these results suggest that response learning is truly facilitated in this group of individuals.

In light of experiments carried out in rats which have shown that response learning is dominant and even facilitated when the hippocampus is inactivated (Packard and McGaugh, 1996; Schroeder et al., 2002; Chang and Gold, 2003a), we hypothesize that impaired “hippocampus-dependent” place learning may facilitate “striatum-dependent” response learning. Our previous study in individuals with DS revealed severe impairments of high-resolution place learning capacities, but suggested a relative preservation of low-resolution place learning capacities (Banta Lavenex et al., 2015). Our current results confirm the relative preservation of low-resolution place learning capacities, as well as a facilitation of response learning in individuals with DS, as compared to MA-matched TD children. Using the same paradigm as in the current study, we previously showed that severe impairments in low-resolution place learning are accompanied by a large facilitation of response learning in WS (Bostelmann et al., 2017), another genetic neurodevelopmental disorder affecting the hippocampal formation (Meyer-Lindenberg et al., 2005; Meyer-Lindenberg et al., 2006). Interestingly, results from a previous study performed in children with ADHD also suggested that the normal interaction between place and response learning may be altered (Robaey et al., 2016). In their study, children were trained on a virtual 8-arm radial maze that was surrounded by visual cues that appeared in the distance. The task could be solved by employing either a response or a place learning strategy. The strategy used by children during training was assessed in a probe trial without any visible landmarks. Twenty percent of the control children exhibited perfect performance on the probe trial, indicating that they were relying on response learning during the training phase. By contrast, 35% of children that exhibited one or more ADHD symptoms exhibited perfect performance on the probe trial, indicating a greater reliance on response learning (Robaey et al., 2016).

Taken together, the findings from these different studies indicate that response learning may be more easily expressed in individuals with a variety of neurodevelopmental learning disorders associated with abnormal hippocampal function. A comparison of the performance of individuals with WS and individuals with DS leads us to further hypothesize that greater impairments in “hippocampus-dependent” place learning may be associated with greater facilitation of “striatum-dependent” response learning.

### Not All Space Is Created Equal

The current findings confirm that spatial memory is not a unitary process. As discussed previously (Banta Lavenex and Lavenex, 2009; Banta Lavenex et al., 2015; Bostelmann et al., 2017), it is critical to recognize that there are different types of spatial learning and memory systems, subserved by different functional brain networks, which may interact and contribute to guiding behavior, and thus impact overall task performance. Consequently, it is necessary to perform a detailed and systematic evaluation of spatial memory processes in order to define a comprehensive and coherent profile of spatial cognitive abilities, which may help to infer the specific cognitive processes and underlying neurobiological substrates that may be impacted or preserved in DS, as well as in other neurodevelopmental or acquired neurological disorders.

In a study by Pennington et al. (2003) utilizing a virtual Morris water maze, evidence of “hippocampus-dependent” spatial memory impairments in DS was inferred from the fact that during a probe trial without the target object, individuals with DS (*n* = 18; 11–19 years old) spent significantly less time searching for the object in the correct quadrant (16% of the duration of the probe trial) than MA-matched TD children (30%; *n* = 18; individually matched to individuals with DS). In a subsequent study, however, Edgin et al. (2010) failed to show a difference in search time between individuals with DS (27%; *n* = 55; 7–38 years old) and MA-matched TD children (21%; *n* = 36). The fundamental features of the task were designed to replicate the features of the original task developed for rats (Morris, 1981). Children used a joystick to navigate in the virtual arena. Each participant completed four visible-target practice trials, after which the target became invisible and the child was instructed to move around the arena until the target was found. After five trials in this condition, the child was presented with a probe trial during which the target would not appear. The child was prompted to continue searching for the target for a total of 90 sec. Although this task may be adequate to demonstrate global impairments in “hippocampus-dependent” place learning following complete hippocampal lesions, as was shown in rats (Morris et al., 1982; Brun et al., 2002), it is not necessarily adequate to reveal the dysfunction of distinct hippocampal regions. Indeed, rats or mice with CA3 dysfunction are able to acquire the task and exhibit clear recognition of the training quadrant (Brun et al., 2002; Nakashiba et al., 2008). Our current findings, together with the results of our previous study (Banta Lavenex et al., 2015), revealed significant variability in the place learning abilities of individuals with DS, which can nevertheless be characterized by largely preserved low-resolution place learning capacities and severely impaired high-resolution place learning capacities. This pattern of results is thus consistent with the absence of significant differences in the performance of the virtual water maze (Edgin et al., 2010), which would only require low-resolution place learning capacities to discriminate the trained quadrant during the probe trial.

Two other studies by Courbois et al. (2013) and Purser et al. (2015) revealed significant impairments in route learning in virtual environments in individuals with DS. Courbois et al. (2013) concluded that individuals with DS were able to learn specific routes, but they were unable to integrate information about these routes into a configurational representation of the environment. Purser et al. (2015) concluded that individuals with DS exhibited a large deficit in route learning, but the exact nature of this deficit was not clearly identified. This kind of representation was also defined by Purser et al. (2015), as configural knowledge, which consists of layout information about an environment that incorporates the relations, including the distance and direction, between features in that environment. These definitions are consistent with the definition of place learning, which refers to an individual’s ability to learn and remember locations in an allocentric spatial frame of reference, in which locations are encoded in relation to other objects or locations in the environment (i.e., in a viewpoint-independent manner), allowing the construction of a cognitive map of one’s environment (Tolman, 1948; O’Keefe and Nadel, 1978; Banta Lavenex et al., 2014). We would like to argue that the characterization of place learning as being “hippocampus-dependent” (O’Keefe and Nadel, 1978) needs to be further qualified to take into account the functions of distinct hippocampal circuits (Rolls and Kesner, 2006; Lavenex and Banta Lavenex, 2013). Our current results, together with the results of Banta Lavenex et al. (2015), suggest that we can already distinguish between (1) low-resolution, topological representations of the environment, and (2) high-resolution spatial representations that include precise metric information. There are certainly a number of other functions associated with distinct hippocampal regions and/or circuits that should be further studied. Thus, it will particularly important to continue investigating different types of “hippocampus-dependent” cognitive processes and not consider possible discrepancies between experimental results as inconsistencies, but rather as useful information regarding the functionality of specific neurobiological substrates subserving these processes.

Accordingly, it is important to think about the implications of using virtual reality paradigms in order to evaluate the ability of individuals with DS to create allocentric representations of the environment. We do not want to argue that virtual reality should never be used, but researchers should be aware of, and discuss, the possible implications of having limited or no access to certain types of information normally available in the real world. For example, when navigating in a real-world environment, participants have access to nearly 130° of visual flow. By contrast, in virtual reality tasks, participants often perceive an atypical reduced point of view. Indeed, computer screens are normally 41 cm wide and displayed directly in front of the subject at a distance of about 61 cm, yielding a field of view of approximately 37° (Tan et al., 2006). Note that newer technologies using, for example, VR goggles or full room displays, may enable a greater immersion in the virtual environment. However, when navigating in the real world, subjects rely not only on visual information, such as landmarks and visual flow, but also on vestibular and proprioceptive information. Altogether, these different inputs contribute to the creation of allocentric representations (Etienne and Jeffery, 2004). Accordingly, removing vestibular and proprioceptive information decreases spatial memory performance in humans (Ruddle and Lessels, 2006). Similarly, the response properties of hippocampal place cells are less specific when vestibular and proprioceptive information is removed, and only visual information is available (Matsumura et al., 1999; Ravassard et al., 2013). It is thus likely that the place learning capacities of individuals with DS in virtual reality paradigms may be negatively impacted by (1) abnormally limited visual information, and (2) the absence of, or more accurately, the presence of contradictory information provided by the vestibular and proprioceptive systems. Consistent with this view, in the study by Courbois et al. (2013), even TD children had serious difficulties solving the virtual reality task, since only five out of nine children could take the shortcut. By contrast, our study provided evidence that a majority of individuals with DS, as well as nearly all MA-matched TD children, were capable of creating a basic allocentric representation of the environment to find one reward location among four possible locations, in a 4 m × 4 m arena, in which participants had access to coherent visual, vestibular and proprioceptive information. Alternatively, and in contrast to previous studies carried out in virtual environments, it would be important to also determine whether individuals with DS are able to build an allocentric spatial representation of the environment, or cognitive map, in absence of visual information, by relying uniquely on self motion-generated vestibular and proprioceptive information.

## Conclusion

Although our previous study showed that high-resolution place learning is severely impacted in individuals with DS, the current study shows that low-resolution place learning may be relatively preserved in these individuals. Consistent with the theory of a competitive interaction between “hippocampus-dependent” place learning and “striatum-dependent” response learning, response learning appears facilitated in individuals with DS, as compared to MA-matched TD children. Altogether, these findings also suggest that the neural pathways supporting high-resolution place learning may be relatively more impacted in DS, whereas the neural pathways supporting low-resolution place and response learning may be relatively preserved.

## Author Contributions

PBL and PL were responsible for the conception and design of the work, acquisition, analysis and interpretation of the data, and drafting of the manuscript. MB was responsible for the design of the work, acquisition, analysis and interpretation of the data, and drafting of the manuscript. LM was responsible for data acquisition. FC and DM were responsible for data acquisition and manuscript drafting. SV was responsible for conception of the work and manuscript drafting.

## Conflict of Interest Statement

The authors declare that the research was conducted in the absence of any commercial or financial relationships that could be construed as a potential conflict of interest.
